# Cerebellar Abiotrophy in Australian Working Kelpies Is Associated with Two Major Risk Loci

**DOI:** 10.3390/genes13101709

**Published:** 2022-09-23

**Authors:** Claire M. Wade, Annie Y. H. Pan, Rosanne M. Taylor, Peter Williamson

**Affiliations:** 1 School of Life and Environmental Sciences, Faculty of Science, University of Sydney, Camperdown, NSW 2006, Australia; 2 Sydney School of Veterinary Science, Faculty of Science, University of Sydney, Camperdown, NSW 2006, Australia

**Keywords:** cerebellar abiotrophy, cerebellar ataxia, genome-wide association, Australian working kelpie

## Abstract

An autosomal recessive form of inherited cerebellar abiotrophy (CA) that is characterized by a degeneration of Purkinje and granule cells in the cerebellar cortex occurs in the Australian working kelpie dog breed. The clinical signs of CA include ataxia, head tremor, motor in-coordination, wide-based stance, and high-stepping gait. Investigation of clinical and pathological features indicated two closely related diseases with differences in age of onset. A genome-wide association study on 45 CA affected and 290 normal healthy Kelpies identified two significantly associated loci, one on CFA9 and a second on CFA20. Dogs homozygous for the risk haplotype on CFA20 (23 dogs) show clinical signs before ten weeks of age. Missense variants in the sixth exon of disruptor of telomeric silencing 1-like (DOT1Lp.R200Q) and in the only exon of Leucine Rich Repeat And Ig Domain Containing 3 (LINGO3p.R359C), both on CFA20, segregate with the associated risk marker which has incomplete penetrance (42%). Affected dogs homozygous for the risk haplotype on CFA9 have later onset ataxia. A missense variant in exon 5 of Vacuole Membrane Protein 1 (VMP1 p.P160Q) on CFA9 segregates as a fully penetrant Mendelian recessive with later-onset CA. Across mammals, the variety of causative loci so far identified as influencing cerebellar disorders reinforces the complexity of the pathways that contribute to cerebellar development and function, and to the pathophysiological mechanisms that may lead to cerebellar ataxia.

## 1. Introduction

Cerebellar cortical degeneration, also known as cerebellar abiotrophy (CA) has been reported in a number of different dog breeds, including Border Collie [1], Lagotto Romagnolo [2], Labrador retriever [3], Brittany spaniel [4], English bulldog [5], Old English sheepdog [6], Gordon setter [6], Finnish hound [7], Boxer [8], and Australian working kelpie (AWK) [9,10]. The main clinical signs of CA in dogs are ataxia with head tremors, a high stepping gait, poor motor coordination and a wide-based stance in the hind legs. The age of onset can vary from a few weeks in breeds such as Coton de Tulear, AWK, and Beagle [9,11,12], but can be up to months in others, e.g., Italian spinone and Scottish terrier [13,14], or have adult onset in the Boxer, Brittany spaniel and Old English sheepdog [8,15,16]. The clinical signs of CA in dogs result from the degeneration of Purkinje cells in the cerebellum, with some reduction in the granule cell layer [17]. Based on past breeding studies, pedigree analysis and segregation analysis, CA has been suspected or confirmed to have an autosomal recessive mode of inheritance in many of the affected dog breeds [4,6,9,13,14,18,19,20,21,22]. The genetic basis of CA has been identified in 15 dog breeds, with five of these breeds being terrier-related breeds [20]. To date, 11 sequence variants in different genes have been reported as the causal variants for CA among 15 dog breeds [6,7,11,12,13,18,19,20,23,24,25,26,27], but the genetic cause in many breeds remains unknown, including the AWK, in which the disease does not segregate with any known variants [28].

In the AWK and Australian Kelpie (AK) (a sub-population of the breed used for exhibition or as pets), CA has been described as a segregating autosomal recessive disease [9] and is reported to affect less than one percent of dogs in the breeds. The disease was recognized for two decades before it was first formally reported in 1989 when three dogs from one litter were identified with variable severity of clinical signs but no progression in severity of clinical signs [9]. Affected dogs displayed ataxia as the main clinical sign, with poor body coordination, head tremors and a high-stepping gait. Clinical signs in some affected Kelpie pups were apparent at five to seven weeks, but not until twelve weeks of age in others [9,10]. Histological analysis of several dogs confirmed a reduction in granule and molecular cell layers with secondary loss of Purkinje cells in severely affected individuals [10].

Identification of risk factors for the disease, including associated genetic markers or causal mutations, will allow informed management decisions for breeders and improve breed health. One genome-wide association study (GWAS) conducted in the Australian working kelpie (AWK) reported an associated region on CFA3, however a causal mutation was not found, and further interpretation is complicated by a breed sub-structure influencing this region [29]. The subjects of the present study were a much larger group of well-characterized affected AWK. The clinical and pathological features of CA are reported in these dogs, and their clinical details utilized for inclusion in a comprehensive genetic analysis.

## 2. Materials and Methods

### 2.1. Samples and Phenotyping

The present study considered 49 CA affected (Table 1) and 360 normal healthy kelpies that were derived from AWK (livestock herding), AK (exhibition/pet-line) and other informally bred kelpies from other sources in Australia (44 cases, 354 healthy) and elsewhere in the world (5 cases, 6 healthy: Germany (10) and Canada (1)). The population genetics of the wider AWK population is described elsewhere [30]. CA affected dogs available to the analysis were predominantly AWK and were referred by veterinarians, the Australian Working Kelpie Council, or through direct contact by the owners.

Affected status was determined by the presence of observed clinical signs consistent with CA, age of onset, neurological exam, and necropsy when possible. Twenty-eight presumptive CA dogs of the forty-nine had clinical checklist or video observations directly assessed by the research team as well as genotyped by Illumina canine genotyping array. A further six array genotyped samples were reported by the owners, or their veterinarians as affected but had no clinical validation by the checklist. The 15 remaining case samples were available from an earlier study [10]. Normal dogs included in the study were reported by owners and breeders as free of the CA clinical signs.

All Kelpie dogs included in the study were enlisted with the written consent of their breeders and owners, in accordance with approved animal ethics protocols (Animal Ethics, University of Sydney, 5928, 2015/902; 2018/1442 and 2021/2005).

### 2.2. DNA Extraction and Array-Based Genotyping

Genomic DNA was extracted from whole blood samples using the phenol-chloroform salting-out method as previously described [28] or the spin column-based nucleic acid purification method according to the manufacturer’s protocol (QIAamp; QIAGEN, Melbourne, VIC, Australia). DNA concentration and purity was measured using a NanoDrop Spectrophotometer (Thermo Scientific, Wilmington, DE, USA). An aliquot of each undiluted stock DNA sample was diluted with MilliQ water to a final concentration of 50 ng/μL and stored at −20 °C.

All dogs were genotyped using Illumina Canine HD SNP arrays, assaying 172,155 or 220,853 markers with service provision by GeneSeek Inc (Lincoln, NE, USA) according to manufacturer protocols.

### 2.3. Genome-Wide Association Analysis

The genotyping data were quality filtered using the PLINK software suite [31]. SNPs with minor allele frequency of less than 5% (--maf 0.05), significant deviation from Hardy–Weinberg equilibrium (--hwe 0.0005) or missing genotype calls of greater than 10% (--geno 0.1) were removed from the subsequent GWAS. Population stratification was assessed using the multi-dimensional scaling option in PLINK (--cluster --mds-plot 3). Outlier animals from the stratification analysis were removed.

Standard case–control association analysis was applied, and association probabilities were subjected to multiple test correction (--assoc --adjust) and permutation (--mperm 1,000,000). Variants with genome-wide corrected Bonferroni and permuted probabilities of lower than 0.05 were accepted as significant. Where multiple associated markers were co-located, risk haplotypes were identified by manual phasing in case animals.

### 2.4. Whole Genome Sequencing for Mutation Detection

Three AWK procured in relation to the current study (one dog exhibiting symptoms of CA, its dam, and a sib without symptoms at the time of observation) were subjected to whole-genome sequencing (WGS) on the Illumina HiSeq 2500 platform (Illumina, San Diego, CA, USA). The 250 base-pair (bp) paired-end DNA libraries for the samples were prepared using the TrueSeq PCR-free kit and sequenced by a centralized core facility (Ramaciotti Centre for Genomics, University of New South Wales, Australia). A further five whole genome sequences from unrelated healthy AWK were obtained by collaboration that included direct observation by two registered veterinarians and were subsequently included as controls in this study. The additional Kelpies were sequenced with 100 base paired end reads on Illumina HiSeq 2000 (Illumina, San Diego, CA, USA) at the same facility.

Whole genome DNA sequences from the eight sequenced Kelpies were aligned in accordance with the Genome Analysis Toolkit (GATK) best practice protocol [32]. Sequencing reads were aligned as pairs to the UU GSD1.0 reference sequence [33] using the Burrows-Wheeler Alignment tool with default parameters [34]. PCR duplicates were marked and removed using the Picard toolkit. Realignment and recalibration was performed using the GATK pipeline [32,35].

Local realignment was performed around insertion–deletions, and base quality scores were recalibrated with the Genome Analysis Tool Kit (GATK, version 3.6.0) [32,35]. Indexing of the reference sequence and alignment (BAM) files was performed with SAMtools (version 0.1.19) [36]. SAMtools mpileup [36] was used to observe regional sequence coverage and to call variants from the aligned sequences. 

The Variant Effect Predictor (VEP) software [37], and the Variant Annotation Integrator [38] were used to ascertain function for variants called by the aforementioned pipeline. One large structural variant was directly observed from both missing calls in the Illumina Canine HD array and the WGS data. 

Genotype frequencies for favored candidate functional variants were observed in two public data genotyping resources [39,40]. Where required for presentation purposes, variant locations were transferred between canine reference assemblies using the University of California Santa Cruz LIFTOVER tool. All chromosomal locations are reported relative to the UU GSD1.0 assembly [33].

### 2.5. Histopathology

Samples of cerebellum tissue were collected from dogs immediately after euthanasia (*n* = 12) (performed by referring veterinarians or at the University of Sydney Veterinary Teaching Hospital). Brain samples were assessed for histopathological cerebellar structure. Briefly, where the whole cerebellum was available, mid-saggital full thickness blocks were selected from the cerebellar hemispheres, vermis and flocculonodular lobes providing samples of cerebrocerebellar, spinocerebellar and vestibulocerebellar regions. Standard histochemical staining techniques such as haematoxylin and eosin and luxol fast blue were used to detect lesions, abnormalities, and demyelination in formalin-fixed tissue sections.

## 3. Results

### 3.1. Genome-Wide Association

**Figure 1:**
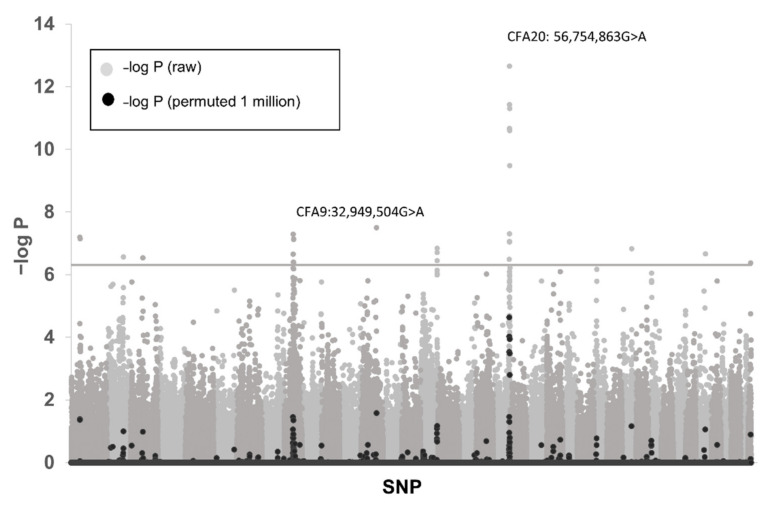
Manhattan plot of genome-wide association with 45 CA affected kelpies and 290 controls. Negative log of raw CHI-squared probabilities is shown in grey, and permuted (1 million permutations) values are shown in black. The most significant associations were identified on CFA20 and CFA9 corresponding with two different disease phenotypes in the data. The notional cut-off for significance (P_raw_) is indicated by a grey line (−log P = 6.3).

The filtered GWAS data consisted of 109,004 SNPs passing quality criteria. After data reduction for population stratification, 45 cases and 290 controls remained in the analysis. A cluster of European dogs (including four cases) was excluded, as were dogs from the AK sub-population that had no cases in these data. Twenty-seven markers passed genome-wide significance after multiple test correction (Bonferroni) (Table 2). Of the 27 markers associated with genome-wide significance, 16 occurred at two loci: CFA9 and CFA20 (Figure 1, Table 2) and of the top 10 associated markers, nine occurred at these two loci. Genotypes for 335 dogs in the association analysis and the four previously excluded case dogs at the top loci are shown in Table S1.

On CFA9, six markers (Table 2) met genome-wide significance after Bonferroni correction. From array genotypes, we observed homozygosity for the most associated risk-associated allele on this chromosome: BICF2G630835610 CFA9:32,949,504G>A, (p_genome_ 5.13 × 10^−^^8^) in 11 CA cases and no normal dogs. All but two of the cases affected by homozygosity for risk-associated array-markers at this locus exhibited the later-onset form of CA. Of the remaining two animals ID 6394 had an early onset of disease (8 weeks) while ID 6111 had unknown age of onset (Table 1). Excluding animals that were homozygous for the associated risk alleles on CFA20, CA clinical penetrance for homozygous risk genotype at the CFA9 was 100% (11/11 affected).

An extended region on CFA20 accounted for a further 23 CA cases, including all remaining Australian dogs with verified diagnosis. Four European littermates with positive diagnosis of CA remained unexplained. The broader CFA20 region was characterized by ten genome-wide significant SNPs within 2.49 megabases (Mb): CFA20: 55,914,798–58,480,589 (span defined by 500 kb flanks for the first and last significantly associated markers) (Table 2). Of array SNPs, the two most highly associated were BICF2P1109624, CFA20:56,754,863G>A (p_genome_ = 2.41 × 10^−^^8^) and TIGRP2P278295, CFA20:56,781,667C>A, (p_genome_ = 4.05 × 10^−^^7^). Individual genotypes are provided in Table S1. Excluding animals accounted for by CFA9, penetrance for homozygous risk genotype at CFA20:56,754,863G>A was 42% (19/45 with homozygous risk genotype observed to be affected) among dogs genotyped by array. All of eight dogs that were homozygous across the entire CFA20 region were affected. One case dog (ID 6111), unobserved by the current clinical team, was homozygous for risk genotypes at both CFA9 and CFA20 risk loci. Fifteen genotyped dogs of the forty-nine described as cases were not homozygous at either CFA9 or CFA20 region. However, of 26 CA dogs with both clinical and/or histopathological confirmation of CA by the research team together with genotyping array results, all other than four European pups born within the same litter were homozygous for risk alleles at either of or both the CFA9 or CFA20 associated sites (Table 1 and Table S1). 

Including consideration of significantly associated loci on other chromosomes identified in the primary analysis did not improve disorder prediction (Table 2). One associated locus on CFA16 is close to the K-locus (CBD103) that codes for the tan-marked coat color that externally typifies the AWK sub-population [29,41].

### 3.2. Clinical Findings and Histopathology

Analysis of brain samples from five affected animals that were homozygous for the CFA9 risk locus and seven animals that were homozygous for the CFA20 risk locus are shown in Table S3. Clinical checklist and video observations of all research-team assessed dogs are shown in Tables S4 and S5. In the observational data, the ages of onset appeared to occur as two clusters, one in the range of 4–10 weeks, and another with a later age of onset (>4 months).

Histopathology shows that affected animals regardless of age of onset exhibit granule cell loss, regional Purkinje cell loss and activated astrocytes. However, the histopathological features are distinct between animals affected with the early-onset disease associated with homozygosity at CFA20 and those affected with the later-onset disease that were homozygous at CFA9. The early-onset group (CFA20) demonstrate abnormality of foliar development in the cerebellar cortex, and a relatively reduced molecular layer (ML). Dogs that were homozygous for the CFA9 locus had normal foliar development, later disorder onset, and demonstrated axonal spheroids in their white matter tracts. Other abnormal features were occasionally observed in both risk groups (Table S3).

### 3.3. Sequence Alignment and Variant Calling

While sequence alignments and variant calling were carried out on a genome-wide scale for the eight sequenced dogs, analysis for the purpose of mutation detection was restricted to risk associated regions on CFA9 and CFA20. Regions of investigation considered 500 Kb beyond the first and last associated marker in each region (CFA9: 32,419,297–35,242,435; CFA20:55,914,798–58,480,589). For the eight whole genome sequenced dogs, 7,848 and 10,602 variant sites were identified that varied from the reference allele in the identified regions of association (CFA9 and CFA20, respectively). 

Based on genotypes at the most associated marker on CFA20 (Table S2), three sequenced dogs—full-sibs 6350 (control) and 6359 (case), plus unrelated 639, were homozygous for the risk haplotype and 6348 (dam of sibs) was heterozygous. Note that array variant genotypes are shown in the top-orientation in Table S1, and variants genotyped by sequencing are shown in the forward orientation relative to UU GSD1.0 in Table S2.

### 3.4. Functional Annotation of Variants

Details of variants identified as potentially functional are shown in Table 3. Regions are described in more detail below.

CFA9: From the array variants called on the eight sequenced dogs (Table S2), 6350 (sibling of early-onset case), and 635 (unrelated control) were expected be heterozygous for risk (enabling detection of putative functional variants in the heterozygous state) while other sequenced dogs were predicted to be homozygous low-risk. Only variants present in the two heterozygous dogs and absent from the remaining animals were considered as candidates. Six variants throughout the region of investigation were predicted to cause putative functional changes as a result of missense or splicing alterations of genes. Of these, one variant was predicted to disrupt a start-site in a retrotransposed pseudogene. The remaining five variants were: CFA9:33,714,212A>T Proline-rich 11 (PRR11) missense (rs24562265); CFA9:33,725,826C>T SMG8 Nonsense Mediated MRNA Decay Factor (SMG8) missense (rs851657894); CFA9:33,780,316G>A splice region Glycerophosphodiester Phosphodiesterase Domain Containing 1 (GDPD1) (rs24562703); CFA9:34,218,228C>A Vacuole Membrane Protein 1 (VMP1) missense (new) and CFA9:34,322,737G>T splice region Tubulin Delta-1 (TUBD1) (new). Of the identified putative functional variants, only CFA9:34,218,228C>A (VMP1 p.P160Q) segregated in linkage-disequilibrium (LD) with the expected risk (Table S2). At this variant, automated calling initially allocated ID 634 as homozygous risk, but manual inspection of the alignment reveals the presence of a reference allele resulting in a heterozygous call. Thus, the VMP1 p.P160Q variant was favored as the best functional candidate for this locus. Both the PolyPhen-2 and the Variant Effect Predictor (VEP) tools [37,42] predict this amino acid change to have a damaging effect on the protein function, with a score of 0.994 (out of a maximum score of 1) for PolyPhen-2 (sensitivity: 0.69 and specificity: 0.97) and a SIFT score of 0 for VEP. The variant allele was unobserved in two comprehensive canine genotype resources [39,40] suggesting that it may be unique to this population (Table S6).

CFA20: Five putatively functional variants segregate among the sequenced dogs in the region of association CFA20:56,414,798–57,980,589 (Table 3). These include four missense variants: ZNF778.p.N11S, CFA20:56,749,717A>G (rs8708774); ZNF77.5.p.G26E, CFA20:56,750,586G>A (rs8708768); LINGO3.p.R359C, CFA20:57,228,029C>T (new); DOT1L.p.R4Q, CFA20:57,306,977C>T (new); and a 12.7 kilobase intronic deletion in Zinc Finger Protein 77 isoform 5 (ZNF77.5) CFA20: 56,731,656–56,744,321DEL (new). Among the dogs with WGS, the case dog (ID 6359) was homozygous for the risk alleles at all five putative functional variants. 

Among dogs homozygous for most associated array marker on CFA20, one putative functional variant CFA20:56,836,520C>T in the gene DOT1 like histone lysine methyltransferase (DOT1Lp.R4Q) differentiated unrelated control (ID 639) and case (ID 6359) that were otherwise homozygous for risk array markers throughout the region (Table S2). The sibling (ID 6350) of early-onset case dog (ID 6359) had insufficient call quality for genotype prediction, though two sequencing reads both showed the risk allele, so this animal was at least heterozygous for risk. The dam ID 6348 was uncalled but manual inspection of the alignment revealed multiple reference allele reads and a single read with the risk allele suggesting heterozygous genotype at the variant. 

For the remaining four variants in genes Zinc Finger 778 (ZNF778), Zinc Finger Protein 77 isoform 5 (ZNF77.5) and Leucine Rich Repeat and Ig Domain Containing 3 (LINGO3), three individuals were homozygous for risk: ID 6359 (case), ID 6350 (normal sibling) and ID 639 (unrelated control)) while the dam (ID 6348) and one unrelated control (ID 636) were heterozygous (Table S2). Closer manual inspection of the variant in ZNF778 using the University of California Santa Cruz (UCSC) genome browser revealed that it was likely intronic.

Among sequenced dogs, the LINGO3p.R359C variant segregated in strong LD with the risk allele of the associated marker: BICF2P1109624, CFA20: 56,754,863G>A. Among sequenced dogs, the DOT1Lp.R200Q mutation differentiates case and control animals that are homozygous for the risk allele of the array marker. Both PolyPhen-2 and Variant Effect Predictor (VEP) [37,42] predict that the DOT1Lp.R200Q mutation is probably damaging with a score of 0.979 (sensitivity: 0.76; specificity: 0.96). The LINGO3 variant is similarly damaging (Polyphen-2 severity score of 1).

Both the LINGO3p.R359C and DOT1Lp.R200Q risk variant alleles were observed at low frequencies in the public variant resources [39,40]. Neither data resource included samples from the Kelpie breed, however other livestock working dog breeds (Australian cattle dog, border collie, Entlebucher cattle dog, Entlebucher sennenhund, Leonberger and Saint Bernard) segregated the DOT1Lp.R200Q allele (only heterozygotes observed) and two breeds (Mallinois, Kerry Blue terrier) segregated the LINGO3 variant (only heterozygotes observed) (Table S6). The propensity for CA in these breeds is currently unreported.

## 4. Discussion

This study identified two distinct clinical and pathological features of CA in Australian working kelpies. These were associated with two separate genomic regions that explain symptoms as recessive inherited traits in 32 of 49 putatively affected dogs and in 22 of 22 Australian dogs with confirmed CA diagnosis. The two individual association regions were shown to be associated with alternative clinico-pathological features of the disease. An early-onset CA form is observable within a few weeks of birth and in some cases is seen within days of birth. This form affects dogs that are homozygous for a risk marker on CFA20. A second region of association, identified on CFA9, is associated with a later-onset form of CA that is not usually diagnosed until the dogs are 4–6 months or older. Excess relationship relative to the wider cohort was unobserved in either explained CFA20 risk-homozygous cases, or CFA9 risk-homozygous cases.

Risk of early-onset CA was positively associated within a region CFA20:55,914,798–58,480,589. In this region two putative functional variants are predicted to be damaging, a C/T substitution in the only exon of LINGO3, NM_001101391p. R359C and a C/T substitution in the sixth exon of DOT1Lp.R200Q (Table 3 and Table S2). Of the two variants, the DOT1Lp.R200Q segregated in stronger LD with the observed phenotypes among the dogs with WGS. By the most associated array marker dog ID 636, which is a highly competitive working dog with normal phenotype, is predicted to be homozygous for risk. This dog is homozygous for the risk allele at LINGO3 but is heterozygous for risk at DOT1L.The observation of ID 636 at this locus is prescriptive of the clinical penetrance of most associated array marker BICF2P1109624, CFA20:56,754,863G>A (p_genome_ = 2.41 × 10^−^^8^) more broadly, such that only 42% (19/45) of genotyped individuals that were homozygous for the risk genotype were identified as affected. Application of genetic testing for the LINGO3 variant by a commercial testing facility (Dog Breeding Science, Sydney, Australia) suggests that of the first 224 dogs tested for the LINGO3 variant, 12 were predicted to be affected, and of these nine were symptomatic (https://breeding.dog/index.php?about=cam300 (accessed on 1 September 2022)). The DOT1L variant is yet to be validated but offers promise for improved test penetrance.

The clinical presentation of the early-onset CA phenotype appears to span a range of severities. In clinical observations (Tables S4 and S5) ages of onset for this phenotype varied from severely affected pups with “generalized ataxia” at 4 weeks of age, through to those that only showed “occasional loss of balance” at 8 weeks. Anecdotal evidence from owners who have kept mildly affected pups suggests that the pups learn to compensate with the disorder, or at least the disorder does not worsen. While animals with minor symptoms may escape diagnosis, we cannot exclude that multiple variants in the region contribute collectively to clinical outcome. Two unexplained cases (ID 6182 and ID 6183) were homozygous for rare alleles in the vicinity of the gene Ceroid Lipofuscinosis 8 (CLN8) suggesting that other low frequency disorders with neurological phenotypes may account for at least some of the remaining unexplained cases in this population. Unexplained cases exhibited no obvious interaction effect between the two main loci.

The DOT1L gene mediates the methylation of histone H3 at position lysine 79 (H3K79). The conditional knockout of DOT1L in mouse cerebellar granule cells (Dot1l-cKOAtoh1) reduces the size of the external granular layer and there are fewer precursors of granule neurons and a smaller cerebellum [43]. Mutant mice showed mild ataxia in motor behaviour tests. The same study showed that Purkinje cell-specific conditional knockout mice displayed no obvious phenotype [43].

Genome-wide significant association identified a second significant region of association CFA9:32,419,297–35,242,435 (UU GSD1.0). The most associated array marker: BICF2G630835610 CFA9:32,949,504G>A, (p_genome_ 5.13 × 10^−^^8^) segregated in perfect LD with a missense variant in the fifth exon of VMP1 NM_030938p.P160Q. Animals homozygous for the risk allele were universally CA affected with 9/11 experiencing a later-onset CA and one with onset at 8 weeks of age (Table 1). One further CA affected individual (ID 6111) had unknown age of onset. A commercial testing facility (Dog Breeding Science, Sydney, Australia) suggests that of the first 286 dogs tested for the VMP1 variant, five were predicted to be affected, and of these all were symptomatic (https://breeding.dog/index.php?about=cam300 (accessed on 1 September 2022)). Homozygosity for the VMP1 variant test has been observed in phenotypically affected working border collies tested by the laboratory.

Animals affected by CA and homozygous for risk alleles at either of the identified CFA20 or CFA9 loci are characterized by the distinctive loss of Purkinje cells in the cerebellum, granule cell loss and the presence of activated astrocytes (Table S3). In the fully penetrant Mendelian recessive later-onset form that segregates with the VMP1 coding variant the cerebellum has normal foliar development. However, axonal spheroids are observed in white matter tracts of the cerebellum as a distinctive histological feature upon histopathological examination of cerebellar tissue. Dogs that are homozygous for the CFA20 risk haplotype have abnormal foliar development, frequently have a reduced molecular layer, and show irregular spacing between the Purkinje cells. The differences in clinical presentations align with likely differences in underlying molecular pathogenesis connecting the associated genomic regions to the biological processes of cerebellar development.

VMP1 is one of the many autophagy-related genes (ATGs) that are necessary for the formation of autophagosome in mammalian cells [44]. A previous study showed that binding of Beclin-2 to Beclin-1 inhibits autophagy in the absence of the VMP1 protein, and expression of VMP1 protein results in dissociation of the Beclin-1/Beclin-2 complex that enables the autophagy process to commence [44,45]. A dysfunctional VMP1 protein may prevent the dissociation of the Beclin-1/Beclin-2 interaction and inhibit the formation of autophagosomes in mammalian cells. Knock-out mouse models disrupting autophagosomes result in progressive motor in-coordination and balance deficits, along with loss of Purkinje cells, axonal swellings, and an accumulation of ubiquitin inclusion bodies [46]. The clinical signs and pathology observed resemble those of the dogs affected by later-onset CA in the present study, in which Purkinje cell death and axonal spheroids are key features.

A missense mutation in exon 1 of the autophagy-linked GTPase (RAB24) gene is known to cause a later-onset form of cerebellar ataxia in Old English sheepdogs and Gordon setters [6]. An early-onset cerebellar ataxia in Finnish hounds is linked to a homozygous missense mutation in the sel-1 suppressor of lin-12-like (SEL1L) gene [7]. The SEL1L gene is a part of the endoplasmic reticulum-associated protein degradation machinery responsible for the ubiquitin-dependent degradation of misfolded endoplasmic reticulum proteins. In Lagotto Romagnolo dogs, a missense mutation in the autophagy-related protein 4 homolog D (ATG4D) gene also on CFA20 is responsible for a vacuolar storage type of cerebellar ataxia [24]. These three naturally occurring canine cerebellar ataxias further reinforce the importance of autophagy in Purkinje cell survival.

## 5. Conclusions

The current work identifies distinct forms of CA in Australian working kelpie dogs, and two novel genomic regions affecting risk. Three putative functional variants are favored (CFA9 VMP1p.P160Q, CFA20 DOT1Lp.R200Q, and CFA20 LINGO3p.R359C). Unavailability of case samples from AK prevented the validation of the associated loci in that sub-population, although the common ancestry of the two sub-populations would predict that the same phenotypes and likely causal variants would be observed in that cohort. The Australian working kelpie maintains an open registry and genes are shared among active livestock herding dogs bred outside of the formal registries meaning that the same disorders may manifest in crosses with other herding breeds such as working border collies and mixed breed herding dogs. Common ancestry of the CFA20 putative functional variant DOT1Lp.R200Q was observed with other livestock herding breeds with genotyping resources in the public domain.

The genes identified in this study add to the body of knowledge of cerebellar development and function.

## Figures and Tables

**Table 1:** Characteristics of 49 case samples including their genotypes at risk loci identified by GWAS. Risk genotypes for each locus shown in bold type.

ID	Sex	Onset Age	Onset-Type	Country	Video (Table S5)	Neurological Exam (Table S4)	Cerebellar Abiotrophy Diagnosis Supported	Homozygous Risk Allele	Genotype CFA20 BICF2P1109624 (AA Risk)	Genotype CFA9 BICF2G630835610 (AA Risk)	Genotype CFA1 TIGRP2P904 (AA Risk)	Genotype CFA13 BICF2P336113 (AA Risk)	Clinical Assessment Source
1827	F	4 weeks	early	Australia		Y	Y	Y	**A A**	G G	G G	G G	Owner-checklist
3596	M	6 weeks	early	Australia				Y	**A A**	A G	G G	A G	Owner
6273	M	6 weeks	early	Australia	Y		Y	Y	**A A**	G G	A G	G G	Veterinarian
6274	F	6 weeks	early	Australia	Y		Y	Y	**A A**	G G	A G	**A A**	Veterinarian
6317	M	4 weeks	early	Australia		Y	Y	Y	**A A**	G G	G G	A G	Neurologist
6343	F	6 weeks	early	Germany	Y		Y		G G	G G	G G	A G	Checklist
6344	M	6 weeks	early	Germany	Y		Y		G G	G G	G G	A G	Checklist
6345	M	7 weeks	early	Germany	Y		Y		G G	A G	G G	G G	Checklist
6346	M	7 weeks	early	Germany	Y		Y		G G	G G	G G	A G	Checklist
6359	F	6 weeks	early	Australia		Y	Y	Y	**A A**	A G	G G	G G	Neurologist
6360	F	6 weeks	early	Australia		Y	Y	Y	**A A**	**A A**	A G	G G	Owner
6369	M	6 weeks	early	Australia		Y	Y	Y	**A A**	G G	G G	G G	Owner-checklist
6370	M	6 weeks	early	Australia	Y		Y	Y	**A A**	G G	G G	G G	Owner-checklist
6376	M	7 weeks	early	Australia				Y	**A A**	G G	G G	A G	Owner
6386	F	7 weeks	early	Australia		Y	Y	Y	**A A**	A G	G G	A G	Owner-checklist
6387	F	5 weeks	early	Australia				Y	**A A**	A G	A G	A G	Owner
6390	F	6 weeks	early	Australia				Y	**A A**	G G	G G	G G	Owner
6394	M	8 weeks	early	Australia		Y	Y	Y	G G	**A A**	G G	G G	Owner-checklist
6395	M	8 weeks	early	Australia				Y	**A A**	G G	A G	G G	Owner
NH3	F	4 weeks	early	Australia		Y	Y	Y	**A A**	G G	A G	A G	Owner-checklist
NH4	F	5 weeks	early	Australia		Y	Y	Y	**A A**	G G	A G	A G	Owner-checklist
6205	M	8 months	late	Australia	Y	Y	Y	Y	G G	**A A**	G G	G G	Veterinarian
6263	M	5 months	late	Australia	Y	Y	Y	Y	G G	**A A**	A G	A G	Checklist
6363	F	16 weeks	late	Australia		Y	Y	Y	G G	**A A**	A G	A G	Checklist
6364	M	16 weeks	late	Australia		Y	Y	Y	G G	**A A**	G G	G G	Checklist
6365	F	6 months	late	Australia		Y	Y	Y	G G	**A A**	G G	A G	Checklist
6368	F	12 months	late	Australia		Y	Y	Y	G G	**A A**	G G	A G	Checklist
6374	F	18 months	late	Australia		Y	Y	Y	A G	**A A**	G G	A G	Checklist
6375	F	16 weeks	late	Australia		Y	Y	Y	A G	**A A**	G G	A G	Checklist
6389	F	15 weeks	late	Australia				Y	A G	**A A**	G G	G G	Owner
6275	F	24 weeks	late	Australia		Y	N		G G	G G	G G	G G	Veterinarian
6266	M	10 months	late	Canada		Y	N		0 0	A G	A G	A G	Veterinarian
6025	F	unknown	unknown	Australia					G G	G G	G G	G G	Previous
6050	M	unknown	unknown	Australia		Y	Y	Y	A A	G G	A G	G G	Veterinarian
6054	F	unknown	unknown	Australia					G G	G G	A G	A G	Previous
6065	M	unknown	unknown	Australia				Y	A A	G G	A G	A A	Previous
6066	F	unknown	unknown	Australia				Y	A A	G G	A G	A A	Previous
6067	F	unknown	unknown	Australia					A G	A G	A G	G G	Previous
6110	F	unknown	unknown	Australia				Y	A A	A G	A A	A G	Previous
6111	F	unknown	unknown	Australia				Y	A A	A A	A A	A G	Previous
6149	F	unknown	unknown	Australia					A G	A G	G G	G G	Previous
6181	F	unknown	unknown	Australia					G G	A G	A G	A G	Previous
6182	F	unknown	unknown	Australia					A G	G G	A A	G G	Previous
6183	F	unknown	unknown	Australia					A G	G G	A A	G G	Previous
6184	F	unknown	unknown	Australia					A G	G G	A G	A G	Previous
6195	F	unknown	unknown	Australia					A G	G G	G G	A G	Previous
6203	F	unknown	unknown	Australia					A G	G G	G G	A G	Previous
6206	F	unknown	unknown	Australia	Y	Y	Y	Y	A A	G G	G G	G G	Veterinarian
6256	M	unknown	unknown	Australia				Y	A A	G G	G G	G G	Previous

**Table 2:** Characteristics of significantly associated markers after multiple-test correction for mapping CA in Kelpies. Markers significant by both Bonferroni and permutation (1,000,000 permutations) are shown in bold-face type.

CHR	SNP	Base (UU GSD1.0)	Allele 1 (A1)	Frequency of A1 in Affected	Frequency of A1 in Unaffected	Allele 2 (A2)	CHISQ	P	P _Bonferroni_	P_permuted(1,000,000)_
**1**	**TIGRP2P904**	**27279310**	**A**	**0.28**	**0.08**	**G**	**29.24**	**6.40 × 10^−8^**	**0.006974**	**0.04**
**1**	**BICF2S2379011**	**27300794**	**A**	**0.28**	**0.09**	**C**	**29.06**	**7.03 × 10^−8^**	**0.007665**	**0.04**
2	BICF2P394061	63856960	A	0.31	0.11	G	26.45	2.71 × 10^−7^	0.02951	0.10
3	BICF2S23915658	50921021	A	0.29	0.10	C	26.31	2.91 × 10^−7^	0.03167	0.10
9	BICF2G630835610	32949504	A	0.33	0.12	G	29.67	5.13 × 10^−8^	0.005588	0.04
9	BICF2G630835611	32954974	A	0.38	0.15	G	26.87	2.18 × 10^−7^	0.02372	0.09
9	BICF2G630835989	33963736	C	0.26	0.08	A	25.71	3.97 × 10^−7^	0.04326	0.12
**9**	**BICF2G630836291**	**34689620**	**A**	**0.41**	**0.17**	**G**	**29.03**	**7.14 × 10^−8^**	**0.007777**	**0.04**
**9**	**BICF2G630836293**	**34700358**	**G**	**0.41**	**0.17**	**A**	**28.95**	**7.42 × 10^−8^**	**0.008085**	**0.04**
9	BICF2G630836318	34777634	A	0.13	0.41	G	25.65	4.10 × 10^−7^	0.04465	0.13
**13**	**BICF2P336113**	**43940212**	**A**	**0.30**	**0.09**	**G**	**30.61**	**3.16 × 10^−8^**	**0.003439**	**0.03**
16	BICF2P228482	57096165	A	0.40	0.17	G	25.91	3.58 × 10^−7^	0.03903	0.12
16	BICF2P1097507	57109139	A	0.40	0.16	C	27.09	1.94 × 10^−7^	0.02112	0.08
16	BICF2P957000	57402220	A	0.48	0.22	C	27.68	1.43 × 10^−7^	0.01563	0.07
20	BICF2P204406	56017675	A	0.72	0.43	G	26.12	3.21 × 10^−7^	0.03504	0.11
20	BICF2P113598	56247267	A	0.16	0.46	G	28.64	8.70 × 10^−8^	0.009478	0.05
**20**	**BICF2P1109624**	**56283529**	**A**	**0.64**	**0.25**	**G**	**53.81**	**2.21 × 10^−13^**	**2.41 × 10^−8^**	**2.30 × 10^−5^**
**20**	**BICF2P393253**	**56293416**	**T**	**0.63**	**0.27**	**A**	**44.85**	**2.13 × 10^−11^**	**2.32 × 10^−6^**	**0.0003**
**20**	**TIGRP2P278295**	**56309785**	**A**	**0.63**	**0.26**	**C**	**48.27**	**3.71 × 10^−12^**	**4.05 × 10^−7^**	**9.40 × 10^−5^**
**20**	**BICF2P1309051**	**56352043**	**G**	**0.69**	**0.38**	**A**	**29.75**	**4.91 × 10^−8^**	**0.005357**	**0.03**
**20**	**BICF2P331357**	**56704521**	**A**	**0.07**	**0.35**	**G**	**28.58**	**8.98 × 10^−8^**	**0.009784**	**0.05**
**20**	**BICF2P696292**	**57458652**	**A**	**0.36**	**0.11**	**G**	**39.49**	**3.29 × 10^−10^**	**3.59 × 10^−5^**	**0.0016**
**20**	**BICF2P287740**	**57465507**	**A**	**0.36**	**0.10**	**G**	**44.53**	**2.50 × 10^−11^**	**2.72 × 10^−6^**	**0.0003**
**20**	**TIGRP2P278607**	**57512779**	**A**	**0.35**	**0.09**	**G**	**47.7**	**4.97 × 10^−12^**	**5.42 × 10^−7^**	**0.0001**
28	BICF2S22962329	30582750	G	0.37	0.14	A	27.63	1.47 × 10^−7^	0.016	0.07
34	BICF2G630459982	25388891	C	0.32	0.12	A	26.88	2.16 × 10^−7^	0.02359	0.09
39	BICF2G630539070	6537056	A	0.19	0.52	C	25.59	4.22 × 10^−7^	0.04599	0.13

**Table 3:** Genotypes and predicted outcomes of putatively functional variants in two genomic regions associated with cerebellar abiotrophy in Kelpie dogs.

Chromosome	ID	Base (UU GSD1.0)	Reference	6359	6350	6348	634	635	636	639	640	Gene	NCBI	Ensembl ID	Predicted Change
9	rs24562265	33714212	A A	A A	A A	A T	T T	A T	T T	T T	T T	PRR11	NM_018304	ENSCAFT00000027880.5	N|Y (9)
9	rs851657894	33725826	C C	T C	C C	C C	C C	C C	C C	C C	C C	SMG8	NM_018149	ENSCAFG00000017590	T|I (112)
9	rs24562703	33780316	G G	G G	G G	A G	G G	G G	G G	G G	G G	GDPD1	-	ENSCAFG00000017594	splice region
9		34218228	C C	C C	A C	C C	C C	A A	C C	C C	C C	VMP1	NM_030938	ENSCAFT00000078851.1	P|Q (160)
9		34322737	G G	G G	G G	G G	G G	G G	G G	G G	T G	TUBD1	-	ENSCAFT00000062569.1	splice region
20		56731656–56744321	INS/INS	DEL/DEL	DEL/DEL	INS/DEL	INS/INS	INS/INS	INS/INS	DEL/DEL	INS/INS	intronic ZNF77.5	NM_001080404		intronic deletion
20	rs8708774	56749717	A A	G G	G G	G A	A A	A A	A A	G G	A A	ZNF778	NM_001201407	-	N|S (11)
20	rs8708768	56750586	G G	A A	A A	A G	G G	G G	A G	A A	G G	ZNF77.5	NM_001080404	-	G|E (26)
20		57228029	C C	T T	T T	T C	0 0	C C	0 0	T T	C C	LINGO3	NM_001101391	ENSCAFT00000030859.4	R|C (359)
20		57306977	C C	T T	0 0	0 0	C C	C C	T C	C C	C C	DOT1L	NM_0324823	ENSCAFT00000075488.1	R|Q (200)

## Data Availability

Whole genome sequence data for Australian working Kelpies associated with this project can be accessed in the European Nucleotide Archive: https://www.ebi.ac.uk/ena/browser/view/PRJEB53208. BAM files are available in the NCBI Short Read Archive under BioProject accession numbers PRJNA384913, and PRJNA394976.

## Supplementary Materials

The following supporting information can be downloaded at: https://www.mdpi.com/article/10.3390/genes13101709/s1, Table S1. Genotypes of 45 cases and 290 controls for Bonferroni significant markers identified in the genome-wide association; Table S2. Genotypes of whole-genome sequenced Kelpies at array-associated markers and three putative functional variants on CFA9 and CFA20 (forward orientation on UU GSD1.0). Reference allele no shading, heterozygous for risk (light grey), and homozygous for risk (dark grey). Marker identifiers in bold indicate most associated markers on CFA9 and CFA20; Table S3. Histopathology results for cerebellar abiotrophy affected kelpies with confirmed diagnosis; Table S4. Clinical observation symptom checklist of 33 dogs presumptively affected by cerebellar abiotrophy (CA). Table S5. Video symptom checklist of 18 dogs presumptively affected by cerebellar abiotrophy; Table S6. Observed frequencies of three putatively functional variants in public genotyping resources
